# Evaluation of risk factors for treatment failure in canine patients undergoing photoactivated chromophore for keratitis – corneal cross-linking (PACK-CXL): a retrospective study using additive bayesian network analysis

**DOI:** 10.1186/s12917-023-03779-x

**Published:** 2023-11-02

**Authors:** M. E. Kowalska, A. K. Shukla, K. Arteaga, M. Crasta, C. Dixon, F. Famose, S. Hartnack, S. A. Pot

**Affiliations:** 1 Ophthalmology Section, Equine Department, Vetsuisse Faculty, University of Zurich, Zurich, Switzerland; 2 Epidemiology Section, Vetsuisse Faculty, University of Zurich, Zurich, Switzerland; 3 AniCura Vision Vet Veterinary Eye Clinic, Bologna, Italy; 4 Veterinary Vision, Penrith, UK; 5 Clinique Vétérinaire d’Ophtalmologie Spécialisée, Blagnac, France

**Keywords:** Photoactivated chromophore for keratitis – corneal cross-linking, PACK-CXL, Canine, Corneal Ulcer, Infectious keratitis, Additive bayesian network, Directed acyclic graph

## Abstract

**Background:**

Infectious keratitis is a common ophthalmic condition in canine patients. Sequelae can include keratomalacia and corneal perforation, a vision threatening outcome. Photoactivated chromophore for keratitis – corneal cross-linking (PACK-CXL) is a non-surgical, adjunctive treatment method for infectious keratitis. The goal of this retrospective, multicenter study was to determine risk factors for treatment failure following PACK-CXL in canine patients suffering from suspected infectious keratitis. Medical records from four veterinary ophthalmology services were reviewed, and information related to patient demographics, ophthalmic findings, the PACK-CXL protocol used, and epithelialization time was collected and analyzed. Due to the potential for intervariable relationships, an additive Bayesian network (ABN) analysis was performed to evaluate these complex relationships.

**Results:**

Records for 671 eyes (668 dogs) were included in the analysis. Based on the ABN, in the population included here, patients who underwent an accelerated PACK-CXL protocol were less likely to experience treatment failure versus patients treated with a slow protocol. Mutual dependencies between exposure variables were identified by ABN, which would have been overlooked using classical regression. Corneal re-epithelialization time was shortened following PACK-CXL combined with topical medical therapy compared to PACK-CXL alone.

**Conclusions:**

No risk factors associated with treatment failure were identified in the population included in the present study. Canine patients may benefit from the use of accelerated PACK-CXL protocols, especially when combined with topical antibiotics and anti-collagenolytic therapy. The reasons for this apparent positive impact on treatment outcome remain unclear.

**Supplementary Information:**

The online version contains supplementary material available at 10.1186/s12917-023-03779-x.

## Background

Infectious keratitis is a common ophthalmic condition in canine patients, which can be complicated by keratomalacia, or corneal melting. Keratomalacia is characterized by corneal stromal tissue loss, oftentimes rapidly progressive [1]. Therefore, progression of both infectious keratitis and keratomalacia may result in significant corneal scarring and corneal perforation, ultimately threatening vision and retention of the globe. Infectious keratitis is a challenging disease to treat, which is undoubtedly partially due to the complicated interplay between environmental (e.g., client compliance, presence of local pathogens and seasonality), patient (e.g., breed, age, ocular or systemic comorbidities), and treatment (e.g., available treatment options, clinician preference/experience) associated factors. Fortunately, numerous treatment strategies exist to address infectious keratitis in veterinary patients, including the use of corneal cross-linking (CXL) [2], a promising adjunctive, non-surgical treatment approach for infectious keratitis and keratomalacia [3, 4]. This technique refers to the application of a photosensitizing agent to the corneal surface and subsequent activation using an energy source (e.g., UV-A light), thereby creating new chemical bonds within and between collagen and proteoglycan molecules [5–8] in the corneal stroma. Corneal cross-linking has been shown to increase corneal collagen fiber diameter, increasing corneal stiffness and biomechanical stability [7, 9–11], and was initially developed in the late 1990s for the treatment of keratoconus in human patients [12, 13]. It has also been shown to increase the ability of the corneal stroma to resist digestion by collagenolytic enzymes, including collagenase, pepsin, trypsin, and matrix metalloproteinases [7, 8, 14–21]. Additionally, antimicrobial activity of CXL against various bacterial, fungal and parasitic (amoeba) agents has been demonstrated in vitro and in vivo [22–25]. Photoactivated chromophore for keratitis – corneal cross-linking (PACK-CXL) is a term that specifically indicates the use of this modality for the treatment of infectious keratitis in both human and veterinary medicine [2, 4, 14, 26, 27].

In the authors’ experience, the time required to perform a PACK-CXL treatment is typically less than the time required to perform most surgical stabilization techniques, and it may be performed in awake, quiet animals, or in animals that are sedated or under general anesthesia. Therefore, the authors suggest that PACK-CXL might present a superior treatment approach in the management of infectious keratitis and keratomalacia in some canine patients where surgery may not be a viable option, or where topical therapy alone may be insufficient (e.g. in individuals where a prolonged general anesthetic is not recommended due to concurrent systemic comorbidities, in cases where surgical stabilization may be predicted to fail due to an unstable corneal stroma, where stromal loss has not exceeded 50% and surgical stabilization is not yet required, or where client finances prohibit surgical intervention). It is, however, worth noting that, to the authors’ knowledge, there are currently no published, prospective, randomized controlled trials (RCTs) utilizing PACK-CXL as the sole treatment for suspected infectious keratitis in dogs. Thus, the use of this treatment modality as anything other than an adjunctive therapy in combination with conventional treatment methods remains experimental.

Despite the increasing popularity of PACK-CXL for the treatment of infectious keratitis, as suggested by the increasing number of veterinary papers and abstracts being published on this topic, the authors are not aware of any published research evaluating risk factors for treatment failure using PACK-CXL, or evaluating the proportion of treatment success in populations of canine patients that have undergone PACK-CXL therapy.

Evaluation of risk factors is critically important for clinicians to optimize treatment plans and maximize positive treatment outcomes in their patients. Prior identification of risk factors for treatment failure, or success, is also crucial for RCT planning. Unfortunately, assessment of risk factors in infectious keratitis is exceptionally complicated, likely due to the complex interactions between environmental, patient, and treatment factors.

A common approach in risk factor analysis is to perform a classical regression where all potential risk factors in the analysis are termed exposure variables and the outcome of interest is termed the outcome variable. Classical regression is designed to analyze experimental data with a balanced distribution of exposure variables between the compared groups [28], and it performs well in controlled experimental conditions. However, these conditions are not present in most observational studies, where complex, inter-related associations exist between variables. To analyze and understand complex associations, which can lead to the identification of risk factors, an additive Bayesian network (ABN) analysis may be employed [29–32] as an alternative to classical regression.

Additive Bayesian network analysis is based on machine learning, and is a multi*variate* (many outcomes and many variables possible at the same time) extension of classical regression models. The analysis results consist of two components. The first component is structural, resulting in the creation of directed acyclic graphs (DAGs). A DAG can be understood as a map of variables (nodes) and existing associations (arcs). The second component of an ABN consists of a set of parameters belonging to each arc, which represent the estimates of effect sizes, for example, odds ratios (ORs) or regression coefficients.

Currently, risk factors for treatment failure using PACK-CXL in veterinary medicine are unknown. The objectives of this exploratory retrospective study were to: (1) Explore associations between variables in cases of suspected infectious keratitis treated with PACK-CXL to aid in the generation of hypotheses for future studies. (2) Identify risk factors for treatment failure following PACK-CXL in the cases of suspected infectious keratitis presented here. (3) Establish the proportion of treatment success in the population of dogs studied here who received PACK-CXL as part of their therapy.

## Materials and methods

### Study Design and Patient Population

A retrospective, multi-institutional study was performed. Eyes treated at four ophthalmology services over a combined 10-year time period (France 2013–2021, Italy 2016–2021, Switzerland 2011–2021, United Kingdom 2017–2021) were evaluated for their potential inclusion in the study. Inclusion criteria included dogs that had undergone PACK-CXL therapy for suspected infectious keratitis as determined by the attending ophthalmologist and defined as loss of the corneal epithelium, in addition to stromal loss and/or stromal infiltrates and/or keratomalacia, where the treatment outcome was known. Cases were excluded if these criteria were not fulfilled. The eyes of individuals undergoing bilateral treatment were enrolled as separate cases. Detailed instructions sent to the collaborating ophthalmology services regarding data to be collected are available in Supplementary file 1. The collaborating ophthalmologists classified eligible cases as either treatment failures or successes (criteria for treatment failure or success are included in the “Recorded Data Points” section).

### Recorded data points

#### Primary treatment outcome

failure or success. Treatment failure was defined as a requirement to deviate from the original referral practice treatment plan in order to stop/stabilize keratomalacia. Failure cases were those cases where a change in medical management, a surgical intervention, or second PACK-CXL treatment were required to stabilize the cornea, or where enucleation was pursued. Success was defined as no deviation from the original referral practice treatment plan, with retention of the globe.

#### Secondary treatment outcome

time to complete epithelialization (expressed in days) of the corneal surface defect.

### Potential exposure variables

1) Patient demographics: age, breed, sex, skull type (categorised as: brachycephalic -used as baseline/reference category in the statistical model- or mesocephalic).

2) Ophthalmic co-morbidities were included only if they occurred in the affected eye, and were classified into 4 groups:

- Corneal disease - any individual with a history of corneal disease/corneal disease present, which is not infectious keratitis (e.g., endothelial dysfunction, pigment keratopathy).
- Ocular surgery - any individual with a history of having undergone prior ocular surgery (e.g., phacoemulsification, corneal grafting procedure).
- Nasolacrimal disease - any individual with nasolacrimal disease (e.g., qualitative or quantitative keratoconjunctivitis sicca).
- Other ocular disease - any individual with ocular disease present (e.g., eyelid conformation issues, history of uveitis or glaucoma, distichiasis), but excluding corneal or nasolacrimal disease.

Patients not suffering from any of the afore-mentioned conditions were used as a baseline in the statistical model.

3) Systemic disease was categorized as present or absent: presence was defined as any disease expected to have an impact on ocular health (e.g., endocrinopathies). Absence was defined as no systemic disease or any disease not expected to have an impact on ocular health (e.g., asymptomatic patient with cardiac disease, epilepsy) and was used as baseline.

4) Treatment history: time until referral (expressed in days), antibiotics used prior to referral (“AB prior”: yes/no, baseline: no), steroids used prior to referral (“Steroids prior”: yes/no, baseline: no).

5) Ophthalmic findings: ulcer size (expressed in mm), ulcer depth (expressed in %), keratomalacia at the time of examination (yes/no, baseline: no), hypopyon at the time of examination (yes/no, baseline: no).

6) Initial treatment plan: use of topical medications as part of the referral treatment plan in addition to PACK-CXL (“CXL/topical treatment”: yes/no, baseline: no), use of surgery as part of the referral treatment plan in addition to PACK-CXL (“CXL/surgery”: yes/no, baseline: no). All patients that underwent surgery also received topical medical therapy.

7) The PACK-CXL protocol used, including: fluence (baseline: standard-5.4 J/cm^2^), riboflavin concentration (baseline: standard − 0.1%), acceleration (baseline: fast, defined as any irradiation time below 10 min; additional details in Table 1).

**Table 1:** Protocol classification according to acceleration level

Acceleration category	Irradiation time (minutes)	Irradiation intensity (mW/cm^2^)
Fast	2	45
	3	30
	5	18
Slow	10	9
	30	3

The data was initially evaluated using descriptive statistics [32], followed by advanced statistical modeling which is presented in this article. The primary outcome of treatment failure was analyzed with ABN analysis. The secondary outcome of time to corneal re-epithelialization after PACK-CXL treatment was evaluated using survival analysis. The raw data, metadata and R code for the performed analyses, are available on the Open Science Framework (OSF) repository under osf.io/4hk6s.

### Additive bayesian network (ABN) analysis

**Fig. 1:**
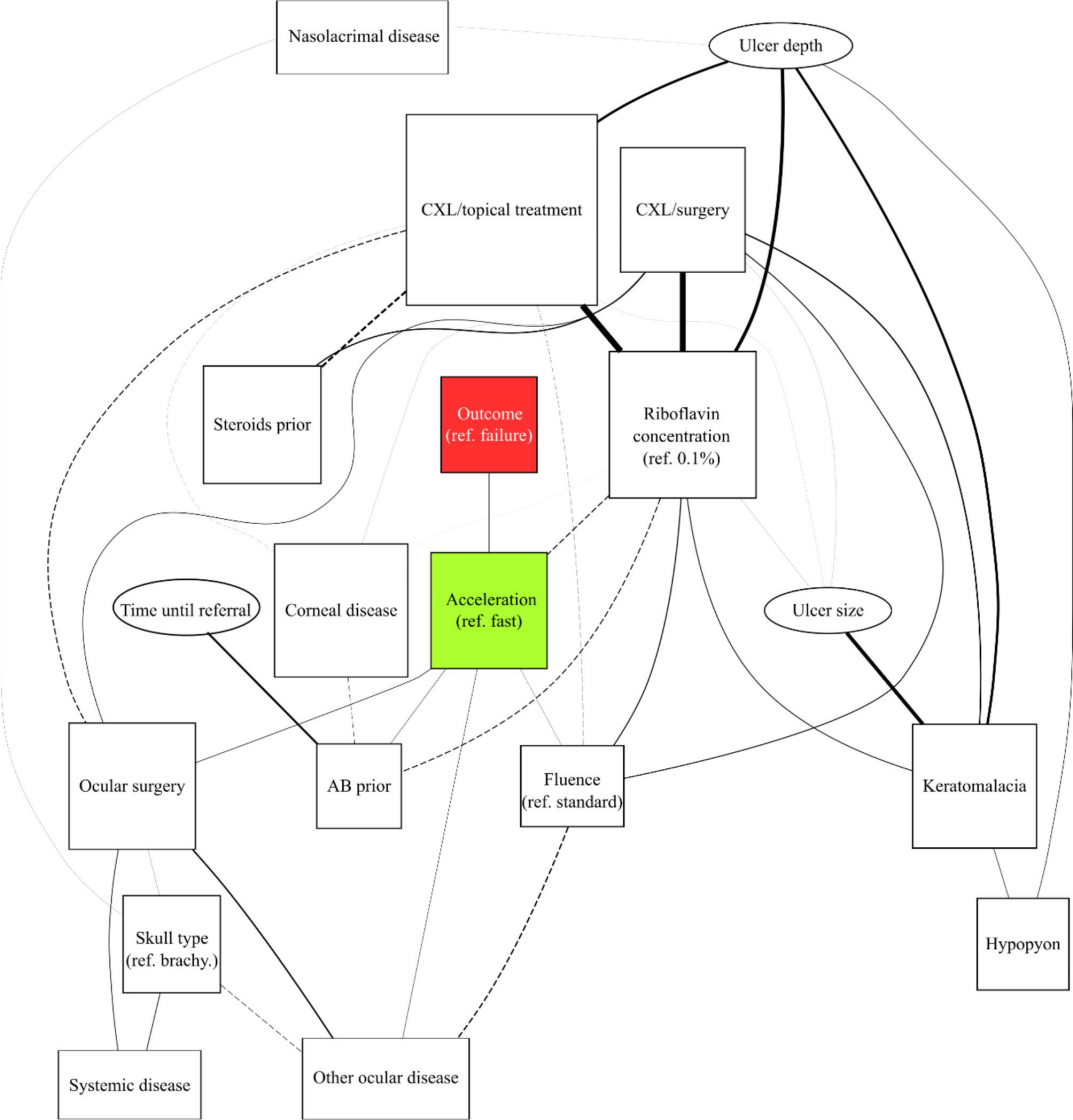
Evaluation of risk factors associated with PACK-CXL treatment failure (primary treatment outcome): Additive Bayesian network (ABN) analysis. The nodes (squares, rectangles and ovals) represent exposure variables, and the red square represents the primary treatment outcome (treatment success/failure). The arcs represent the relationships between variables, with a dashed line indicating a negative association, and a solid line indicating a positive association. Arc thickness represents the strength of support for the association. Oval nodes represent variables with Gaussian distributions. Quadratic nodes represent variables with binomial distributions. For the variables without baseline indicated in the graph, the baseline value was “no”. Acceleration is the only exposure variable directly associated with the outcome. No other variables were directly linked to the outcome, but some variables were indirectly linked to the outcome through acceleration

Eighteen exposure variables (listed above and in Tables 2 and 3 and Figure S1) and the primary treatment outcome, were considered for ABN analysis. Age, sex, and breed, and bacterial culture results were excluded for the following reasons. Age and sex were similarly distributed in both the treatment success and failure groups (Table 4). Analyzing risk factors on a breed level would be very difficult due to a large number of subgroups with a small number of patients per subgroup. The statistical power of such subgroup analyses would be very limited. The authors therefore decided to analyze skull type instead. Finally, bacterial culture results were only available for a subset of patients and were therefore not considered further in this study. The ABN analysis was performed using a two-step approach; details can be found in Supplementary file 2. The strength of each arc was quantified and is reflected in the arc thickness in the DAG (see Fig. 1). The marginal posterior log odds ratios, correlation coefficients, and 95% credible intervals were estimated for each arc from the posterior distribution (Table 5). Additionally, to adjust for a clustering effect due to the multi-institutional nature of this study, “place” (clinic location) was used as a random effect in the ABN. For completeness, and to reveal significant associations selected in a classical approach, the same variables as considered in the ABN were analyzed with a generalised mixed model using the “lme4” package [33], with failure as the outcome, and the place as a random effect. All analyses were performed using R software, version 4.0.2.

**Table 2:** Number of dog eyes in each category after imputation of missing values for exposure variables

ID		Treatment success group n (%) [95%CI]	Treatment failure group n (%) [95%CI]	Total n (%) [95%CI]
	*Patient demographics*			
1	Skull type			
	brachycephalic	406 (90) [86.5 to 92]	47 (10) [8 to 13]	453 (67.5) [64 to 71]
	mesocephalic	199 (91) [87 to 94]	19 (9) [5.5 to 13]	218 (32.5) [29 to 36]
	*Medical history*			
2	AB prior			
	No	190 (87.5) [83 to 91]	27 (12) [8.5 to 17]	217 (32) [29 to 36]
	Yes	415 (91) [88.5 to 94]	39 (9) [6 to 11]	454 (68) [64 to 71]
3	Systemic disease			
	No	581 (90.5) [88 to 93]	61 (9.5) [7 to 12]	642 (96) [94 to 97]
	Yes	24 (83) [72 to 97]	5 (17) [7 to 31]	29 (4) [3 to 6]
4	Steroids prior			
	No	597 (90.5) [88 to 92.5]	63 (9.5) [7 to 12]	660 (98) [97.5 to 99]
	Yes	8 (73) [54.5 to 100]	3 (27) [9 to 55]	11 (2) [1 to 2.5]
5	Corneal disease			
	No	520 (90.5) [88 to 93]	54 (9.5) [7 to 12]	574 (85.5) [83 to 89]
	Yes	85 (88) [82 to 94]	12 (12) [7 to 19]	97 (14.5) [12 to 17]
6	Ocular surgery			
	No	568 (91) [89 to 93]	58 (9) [7 to 11.5]	626 (93) [92 to 95]
	Yes	37 (82) [73 to 94]	8 (18) [9 to 29]	45 (7) [5 to 9]
7	Nasolacrimal disease			
	No	553 (90) [88 to 92.5]	60 (10) [7.5 to 12]	613 (91) [89 to 93]
	Yes	52 (90) [84 to 90]	6 (10) [5 to 19]	58 (9) [7 to 11]
8	Other ocular disease			
	No	457 (91) [89 to 94]	43 (9) [6 to 11]	500 (81) [78 to 84]
	Yes	148 (86.5) [82 to 91]	23 (13.5) [9 to 18]	171(19) [16 to 22]
	*Corneal ulcer parameters*			
9	Keratomalacia			
	No	198 (94) [92 to 97.5]	12 (6) [3 to 9]	210 (31) [29 to 35]
	Yes	407 (88) [96 to 91]	54 (12) [9 to 15]	461 (69) [65 to 72]
10	Hypopyon			
	No	510 (91) [89 to 93]	52 (9) [7 to 12]	562 (84) [81 to 86.5]
	Yes	95 (87) [82 to 93]	14 (13) [7 to 19]	109 (16) [13.5 to 19]
	*Treatment plan*			
11	CXL/topical treatment			
	No	88 (86) [80 to 93]	14 (14) [7 to 20]	102 (15) [13 to 18]
	Yes	517 (91) [89 to 93]	52 (9) [7 to 12]	569 (85) [82 to 88]
12	CXL/surgery			
	No	423 (90) [87 to 93]	47 (10) [7 to 13]	470 (70) [67 to 74]
	Yes	182 (91) [87 to 95]	19 (9) [6 to 16]	201 (30) [27 to 34]
	*PACK-CXL protocol parameters*			
13	Fluence (J/cm^2^)			
	5.4	333 (91) [88 to 94]	33 (9) [6 to 12]	366 (54.5) [51 to 58.5]
	10.8	38 (97.5) [95 to 100]	1 (2.5) [0 to 7]	39 (6) [2 to 10]
	16.2	233 (88) [85 to 92]	31 (12) [8 to 16]	264 (39) [35 to 43]
	≥ 21.6	1 (50) [50 to 100]	1 (50) [50 to 100]	2 (0.5) [0 to 4]
14	Acceleration			
	Fast	569 (92) [90 to 94]	51 (8) [6 to 10]	620 (92) [90 to 94]
	Slow	36 (70.5) [59 to 82]	15 (29.5) [18 to 41]	51 (8) [6 to 10]
15	Riboflavin concentration			
	0.1%	400 (90) [88 to 93]	42 (10) [7 to 12]	442 (66) [62 to 70]
	0.23%	201 (89) [86 to 93]	24 (11) [7 to 15]	225 (33.5) [30 to 37]
	0.25%	4 (100)	0 [0 to 42]	4 (0.5) [0 to 4]
	**Total**	605 (90) [88 to 92]	66 (10) [7 to 12]	671 (100)

AB prior: antibiotics used prior to referral; Steroids prior: steroids used prior to referral. Where data was not imputed, the quantity of missing values is shown. One can interpret the table by comparing the widths of the confidence intervals within the same group (e.g., within the “success group”). When the confidence intervals of the categories (e.g., brachycephalic/mesocephalic) within an exposure variable (e.g., “skull”) do not overlap, this indicates that this particular exposure variable could be a potential risk factor

Data used in the ABN analysis. For the ABN analysis, total fluence was classified into standard fluence (5.4 J/cm^2^) and increased fluence (> 5.4 J/cm^2^); riboflavin concentration was classified into: standard (0.1%) and high concentration (0.23 and 0.25%). Due to unbalanced numbers of subjects in subgroups, the riboflavin carrier type was excluded from the ABN analysis

**Table 3:** Medical history data and continuous variables used in the ABN analysis

ID		Treatment success group median (interquartile range)	Treatment failure group median (interquartile range)	Total median (interquartile range)
	*Medical history*			
16	Time until referral (days)	6 (3–10)	4 (1.6–8.7)	6 (3–10)
	*Corneal ulcer parameters*			
17	Ulcer size (mm)	5 (3–7)	6 (4–8)	5 (3–7)
18	Ulcer depth (%)	30 (30–40)	30 (30–40)	30 (30–40)

**Table 4:** Number of dog eyes after imputing missing values for variables excluded from the ABN analysis

	Treatment success group n (%) [95%CI]	Treatment failure group n (%) [95%CI]	Total n (%) [95%CI]
*Patient demographics*			
Age (in years)	median = 8 (interquartile range 0.4 to 17.5)	median = 7.2 (interquartile range 0.6 to 17.2)	median = 8 (interquartile range 0.4 to 17.5)
Eye			
OS	300 (91) [88 to 94]	29 (9) [6 to 12]	329 (49) [45 to 53]
OD	299 (89.5) [86 to 92]	35 (10) [7.5 to 14]	334 (50) [46 to 54]
OU	4 (67) [29 to 92]	2 (33) [8 to 71]	6 (0.8) [0 to 5]
unknown	2	-	2 (0.2) [0 to 4]
Sex			
female	253 (90) [86 to 93]	28 (10) [7 to 14]	281 (42) [38 to 46]
male	349 (90) [87 to 94]	38 (10) [7 to 13]	387 (57.5) [54 to 62]
unknown	3	-	3 (0.5) [0 to 0.5]
Breed			
Shih Tzu	111(89.5) [84 to 95]	13 (10.5) [5 to 16]	124 (18.5) [15.5 to 21]
French Bulldog	96 (87) [81 to 93.5]	14 (13) [6.5 to 19]	110 (16.5) [13.5 to 19]
Pug	77 (89.5) [83 to 96]	9 (10.5) [4 to 17]	86 (13) [10 to 15]
Other	321 (91.5) [88.5 to 94]	30 (8.5) [5 to 11]	351 (52) [48.5 to 56]
*PACK-CXL protocol parameters*			
Riboflavin carrier			
Dextran	33 (66) [54 to 79]	17 (34) [22 to 47]	50 (7) [3 to 11]
HPMC	255 (94.5) [92 to 97]	15 (5.5) [3 to 8]	270 (40) [36 to 44]
Other	317 (90) [87 to 93]	34 (10) [7 to 13]	351 (53) [48 to 56]
**Total**	605	66	671

**Table 5:** Regression coefficient estimates and 95% Credible Intervals (CrI) with their interpretation and data support

Arc	Coefficient	95% CrI	Interpretation	Support
Treatment outcome –acceleration **	5.31	2.58 to 10.65	OR	0.0647
Skull type– nasolacrimal disease	2.26	1.53 to 5.38	OR	0.0113
Skull type– ocular surgery	2.83	1.32 to 3.90	OR	0.0143
AB prior – time until referral	4.55	3.20 to 6.93	OR	0.1593
AB prior – corneal disease	0.44	0.26 to 0.75	OR	0.0340
AB prior – riboflavin concentration	0.32	0.21 to 0.46	OR	0.0807
AB prior – acceleration	9.66	3.56 to 49.9	OR	0.0305
Steroids prior – CXL/topical treatment	0.0006*	< 0.001 to 0.22	OR	0.1677
Steroids prior – CXL/surgery	678.6	13 to 7,564,675	OR	0.1022
Systemic disease – skull type	4.18	1.92 to 10.46	OR	0.0595
Systemic disease – ocular surgery	5.75	2.2 to 13.5	OR	0.0591
Corneal disease – riboflavin concentration	1.31	0.66 to 2.71	OR	0.0017
Corneal disease – CXL/topical treatment	0.26	0.11 to 0.64	OR	0.0080
Corneal disease – CXL/corneal surgery	2.27	1.07 to 4.47	OR	0.0074
Ocular surgery – acceleration	5.75	2.12 to 14.53	OR	0.0483
Ocular surgery – CXL/topical treatment	0.12	0.04 to 0.25	OR	0.0888
Ocular surgery – CXL/corneal surgery	4.81	2.23 to 11.89	OR	0.0525
Other ocular disease – skull type	0.42	0.26 to 0.65	OR	0.0448
Other ocular disease – ocular surgery	31.9	14.70 to 82.7	OR	0.1181
Other ocular disease – acceleration	5.52	2.76 to 11.65	OR	0.0340
Other ocular disease – fluence	0.16	0.09 to 0.25	OR	0.1106
Ulcer size – riboflavin concentration	0.135	-0.13 to 0.4	Regression coefficient	0.0091
Ulcer size – CXL/topical treatment	-0.362	-0.7 to -0.04	Regression coefficient	0.0051
Ulcer size – CXL/surgery	0.184	-0.06 to 0.43	Regression coefficient	0.0080
Ulcer depth – nasolacrimal disease	0.47	0.2 to 0.74	Regression coefficient	0.0107
Keratomalacia – ulcer size	2.57	2.03 to 3.4	OR	0.2878
Keratomalacia – ulcer depth	1.4	1.17 to 1.72	OR	0.1943
Keratomalacia – riboflavin concentration	3.44	2.13 to 5.90	OR	0.0690
Keratomalacia – CXL/surgery	3.24	1.93 to 5.70	OR	0.1091
Hypopyon – ulcer depth	1.31	1.08 to 1.57	OR	0.0463
Hypopyon – Keratomalacia	3.22	1.89 to 6.17	OR	0.0459
CXL/topical treatment – ulcer depth	1.45	1.12 to 1.94	OR	0.1924
Fluence – riboflavin concentration	9.45	5 to 19.51	OR	0.0832
Fluence – acceleration	2.85	1.41 to 5.58	OR	0.0178
Fluence – CXL/topical treatment	0.00025*	0.05 to 0.28	OR	0.0182
Fluence – CXL/surgery	6.88	3.94 to 12.02	OR	0.0769
Acceleration – riboflavin concentration	0.10	0.02 to 0.27	OR	0.0853
Ribo. concentration – ulcer depth	0.3	0.13 to 0.47	OR	0.2619
Ribo. concentration – CXL/topical treatment	0.0002*	0 to 0.001	OR	0.4744
Ribo. concentration – CXL/surgery	1199.908*	319.8973 to 48776.31	OR	0.4532

Where two binomial variables are in the arc, such as in the case of skull type (brachycephalic/mesocephalic) – nasolacrimal disease (No/Yes), the results should be interpreted as an odds ratio (OR): mesocephalic dogs have 2.26 (95% CI 1.53 to 5.38) times the odds of having nasolacrimal disease compared to brachycephalic dogs

Where one binomial and one continuous variable are in the arc, such as in the case of keratomalacia (No/Yes)– ulcer size (in mm), the results should be interpreted as an OR: a one mm increase in ulcer size increases the odds of experiencing keratomalacia by 2.57 times (95% CI 2.03 to 3.4)

Where one continuous and one binomial variable are in the arc, such as in the case of ulcer depth (%) – nasolacrimal disease (No/Yes), the results should be interpreted as a regression coefficient: the dogs with nasolacrimal disease have deeper corneal ulcers, with a difference of 0.47% ulcer depth compared to dogs without nasolacrimal disease

*If the proportion of animals in a subcategory is small, there is a problem with model convergence, and the same limitation would appear in the classical regression. As a result, estimated values are not reliable and upper or lower limits of the CI are impossible to correctly estimate. Plots of the variable distributions and proportions in the subgroups are available as Supplementary Figure S1

** When the model is adjusted for the factor “place” (clinic location), the effect size between ‘Treatment outcome’ and ‘acceleration’ is reduced to an OR of 1.55 (95% CI from 0.49 to 4.80)

### Survival analysis

The Kaplan-Meier method was used to analyze a time to event outcome (time between PACK-CXL and complete corneal re-epithelialization), and a Cox proportional hazards regression to estimate hazard ratios (HR), after confirming the validity of the proportional hazards’ assumptions (Supplementary file 2). Variables for the model were selected based on the Likelihood Ratio Test and Akaike information criterion (AIC). All considered variables are summarized in Tables S1 and S2. Only dogs that did not receive any form of corneal surgery as a part of the initial treatment plan, or enucleation, were included in this survival analysis. In other words, only eyes for which the epithelialization time could be evaluated were included. R version 4.0.5 with packages DescTools [34], ggplot2 [35], dplyr [36], Gmisc [37], and survival package [38] were used for these analyses.

## Results

Six hundred and eighty four eyes were identified that met the basic inclusion criteria; thirteen eyes were excluded due to insufficient information related to treatment outcome, leaving records for 671 eyes (668 dogs) with three bilaterally treated patients. A comparison of the summary of the original data set (Table S3) with the summary of the data set with the imputed values (Table 2) showed that the two data sets were similar. Percentages presented in the original data set were within the CIs and similar to the percentages of the imputed data, therefore it can be assumed that the missing values are missing at random, and imputation was successful.

### Descriptive statistics

The overall treatment success in the population of dogs in the present study, all of which underwent PACK-CXL treatment, was 90% (95% CI from 88 to 92%). The initial referral treatment plan included PACK-CXL only in 102 eyes (treatment success 86%, 95% CI from 80 to 93%), PACK-CXL and surgery in 201 eyes (treatment success 91%, 95% CI from 87 to 95%), and PACK-CXL and topical therapy in 368 eyes (treatment success 91%, 95% CI from 88 to 94%). Planned surgical procedures performed at the time of referral and concurrently with PACK-CXL included superficial lamellar keratectomy, corneoconjunctival transposition, conjunctival pedicle graft, corneal transplant, and the application of various collagen scaffolds. Detailed information regarding PACK-CXL protocol parameters is displayed in Tables 2 and 4. The median time to referral was 6 days (Table 3). The median age of dogs at the time of treatment was eight years with an interquartile range from 0.4 to 17.5 years. Eighty-five different breeds were represented in the study population, with 67.5% of included subjects being brachycephalic dogs. A similar distribution of age, eye laterality, and sex were noted in both the success and failure groups (Table 4).

Information related to treatment failure was available for 57/66 eyes. The most common rescue intervention following treatment failure was enucleation (25 eyes), followed by corneal grafting surgery (22 eyes) and corneoconjunctival transposition (6 eyes). Signs related to clinical deterioration after PACK-CXL treatment were recorded in 25 cases and included: keratomalacia (10 eyes), corneal perforation (9 eyes), and endophthalmitis (2 eyes). Tables 6 and 7 summarize the recorded data. The percentage of PACK-CXL treated cases that failed treatment differed across the participating clinics: Clinic A: 32.7%, Clinic B: 7%, Clinic C: 4.5%, Clinic D: 11.1%.

**Table 6:** Rescue interventions in PACK-CXL failures

Enucleation	25
Corneal graft surgery	22
Corneoconjunctival transposition	6
Corneal suture	1
Third eyelid flap	1
PACK-CXL	1
Addition of topical serum	1
Missing information	9
**Total**	66

Table presents number of eyes belonging to each category

**Table 7:** Reported signs of clinical deterioration in PACK-CXL failures

Keratomalacia	10
Corneal perforation	9
Endophthalmitis	2
Graft necrosis	1
Infection under graft	1
Stromal bullae and perforation	1
Self-trauma and perforation	1
Missing information	41
**Total**	66

Table presents number of eyes belonging to each category

### Additive bayesian network analysis

The final DAG consisted of 40 arcs and is presented in Fig. 1. Acceleration of the PACK-CXL protocol was the only variable directly associated with treatment outcome (OR 5.31, 95% CI from 2.58 to 10.65), as identified by ABN analysis, which means that acceleration could be a potential “success factor”. After adjusting for the clustering effect of ‘place’, acceleration remained directly associated with treatment outcome, but with a reduced effect size (OR 1.55, 95% CI from 0.49 to 4.80). No direct or statistically significant risk factors for treatment failure were identified.

When both the DAG and the estimated effect sizes (presented in Table 5) were considered, interesting associations could be appreciated since a DAG presents a graphical overview of the associations between considered variables and how they are related. For example, keratomalacia is linked to deeper (OR 1.4) and to larger (OR 2.57) ulcers with hypopyon (OR 3.22). Moreover, dogs with active keratomalacia had an OR of 3.24 for undergoing both PACK-CXL and surgery. As a result of the ABN analysis, it can be appreciated that these patients not only had active keratomalacia, but also larger and deeper ulcers, which may explain the treatment decision.

The exposure variables ‘ulcer size’, ‘ulcer depth’ and ‘AB prior’ (use of antibiotics prior to referral) were not directly linked to the treatment outcome in the ABN analysis; however, these variables were identified as significant in a classical generalised mixed model regression (p values 0.024, 0.045 and 0.032, respectively). As ABN has the capacity to model associations between all entered variables, the associations between ‘ulcer size’, ‘ulcer depth’, ‘AB prior’ and outcome, are suspected to be of an indirect, rather than of a direct nature. The regression analysis results are presented in Table S4 and Figure S2.

### Survival analysis: time to epithelialization

Data from 433 canine eyes was included in the survival analysis. Of those, six eyes were censored due to missing information regarding time to epithelialization. Tables S1 and S2 summarize the considered exposure variables in the included eyes.

**Fig. 2:**
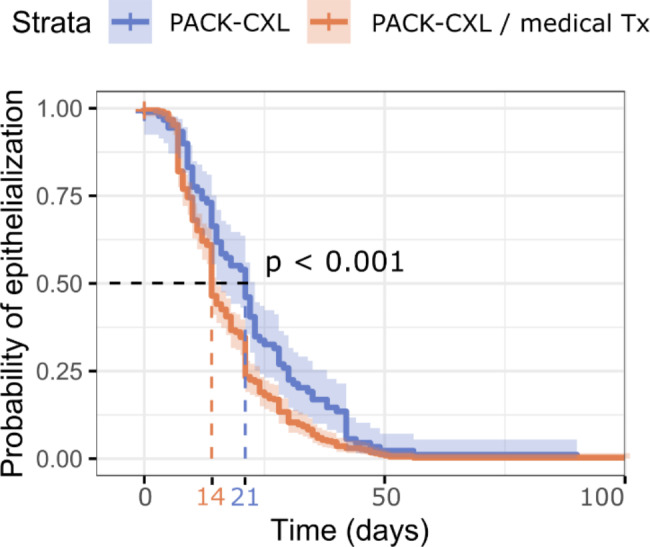
Corneal re-epithelialization time (secondary treatment outcome) of PACK-CXL + topical medical therapy compared to PACK-CXL alone: Kaplan-Meier survival analysis. The median survival time (dashed line) for each group represents the length of time within which the cornea of 50% of patients had re-epithelialized, which was 21 days (95% CI from 16 to 23 days), and 14 days (95% CI from 14 to 15 days), for the groups receiving CXL only, and CXL/medical treatment, respectively (p < 0.001). The straight lines represent the Kaplan-Meier curves and the surrounding dilute colored zones the 95% CIs. Tx: Treatment

The Kaplan-Meier survival curves for the length of time until epithelialization following PACK-CXL or PACK-CXL with additional topical therapy are presented in Fig. 2. The median times to epithelialization were 21 days (95% CI from 16 to 23 days), and 14 days (95% CI from 14 to 15 days), for the groups receiving PACK-CXL only, and PACK-CXL + topical treatment, respectively. The survival curves of these two groups were significantly different (p < 0.001).

**Fig. 3:**
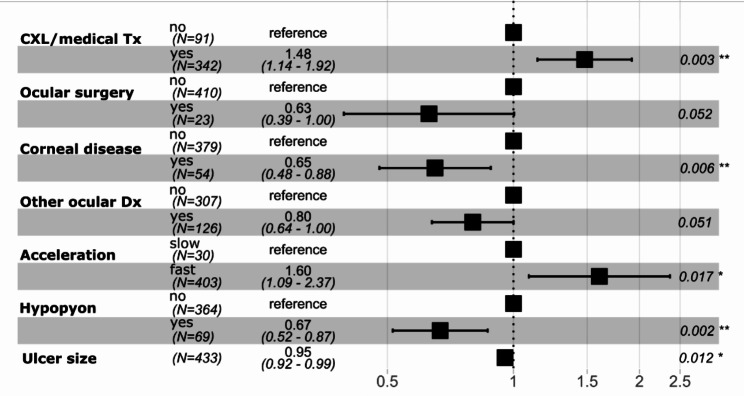
Forest Plot for Cox proportional hazards model. Hazard ratio (HR) marked as black square, 95% CI as horizontal line. A HR < 1 indicates a reduced “risk” of epithelialization compared to the reference group. A HR > 1 indicates a higher “risk” of epithelialization compared to the reference group. Tx: Treatment. Dx: Disease. Variables for the model were selected based on a standard step-wise selection process; details can be found in Supplementary file 2. Based on this approach, age, skull type, time until referral, AB prior, steroids prior, systemic disease, nasolacrimal disease, ulcer depth, keratomalacia, riboflavin carrier, riboflavin concentration, and fluence were not included in the Cox proportional hazard regression

Figure 3 presents the HR based on the Cox proportional hazard regression. Based on this analysis, when comparing two individual dogs with the same baseline exposure variables, a dog that received topical therapy in addition to PACK-CXL is 48% more likely to epithelialize at any given time, compared to a dog that received PACK-CXL only (HR = 1.48, p value 0.003, 95% CI from 1.14 to 1.92). Similarly, a dog that underwent a fast PACK-CXL protocol is 1.60 times more likely to epithelialize versus a dog that received a slow protocol (HR = 1.60, p value 0.017, 95% CI from 1.09 to 2.37). Finally, a dog that had undergone previous ocular surgery, suffered from corneal disease or another ocular disease, and/or hypopyon at presentation to the referral ophthalmology service is less likely to epithelialize at any given time point, compared to a dog without these conditions. Ulcer size was another significant factor influencing epithelialization time. A 1 mm increase in ulcer size led to a 5% reduction of the ability to epithelialize at any given time-point (HR = 0.95, p value = 0.012, 95% CI from 0.92 to 0.99).

## Discussion

The present observational study utilized ABN, a novel method for statistical analysis, instead of a regression model. This statistical methodology was selected since observational studies are typically comprised of interrelated variables, which may limit the utility of regression models. An ABN model does not require researchers to choose a single outcome variable with the remaining variables all being exposure variables. As a result, additional insights may be gained into associations existing between all the variables in the data set, which may not be possible with classical regression. Therefore, ABN may be a more appropriate methodology in observational studies where associations between variables are unknown, or poorly understood.

One purpose of this retrospective study was to identify associations between considered variables and treatment outcome, and subsequently, to identify risk factors for treatment failure in canine patients that had undergone PACK-CXL for the treatment of suspected infectious keratitis. The focus of this study was, therefore, not to compare different protocols (PACK-CXL alone, PACK-CXL + surgery, PACK-CXL + medical management) and establish which protocol was most successful. Instead, this study presents data associated with several recorded variables, including the different treatment approaches employed by various clinicians in the cases included here, and aims to identify if any may be risk factors for treatment failure. A large, comprehensive dataset was collected for this study, including factors suspected to be significant in terms of influencing treatment outcome and factors previously reported in the literature as risk factors for corneal ulcer treatment failure [39]. Several case reports on corneal ulcers in companion animals, focusing on potential predisposing factors and causative infectious agents, have been published [40–43]; however, to the authors’ knowledge, this is the first study evaluating risk factors for treatment failure following PACK-CXL using ABN. Although it has been suggested that brachycephalia and prior use of topical steroids may predispose a patient to corneal ulcers [40, 44], these two variables were not identified as direct risk factors associated with PACK-CXL treatment failure in the ABN in the studied patient population. The reason for this could come from the retrospective nature of the present study, specifically as the result of a selection bias, since the population of dogs selected in the present study may not be representative of the proportion of brachycephalic dogs in the general population. Ultimately, no risk factors for treatment failure were identified in the present study when ABN was utilized.

One exposure variable of interest in the present study was PACK-CXL treatment acceleration. The duration of the irradiation phase during PACK-CXL treatment was divided into a group of fast and a group of slow protocols, with a slow protocol defined as any protocol including at least one 30 min-3 mW/cm^2^ or one 10 min-9 mW/cm^2^ irradiation cycle. Patients undergoing an accelerated (fast) PACK-CXL protocol in the present study were less likely to experience treatment failure, meaning that acceleration could be a potential “success factor”. Effectiveness of accelerated PACK-CXL was previously described in case reports of infectious keratitis in dogs and cats [3, 45], and in human patients [46, 47]. These reports, however, did not include comparison groups. Based on the ABN analysis, acceleration is the only variable directly related to treatment outcome in the present study. When performing a generalized regression mixed model with the same variables as considered in the ABN, the exposure variable ‘acceleration’ as well as ‘ulcer size’, ‘ulcer depth’, and ‘AB prior’ are found to be significant. Since the latter three associations were not identified in the ABN, they are presumably the result of an overfitting, causing the identification of spurious significant associations, i.e., associations that wrongly imply a cause and effect between two variables, a common problem in classical regression analysis. As such, PACK-CXL acceleration was found to be directly linked to treatment outcome in both the ABN and classical regression analysis. The impact of this finding may be significant from a clinical perspective, as the administration of a 2 to 5-minute accelerated protocol to an awake, or sedated patient is more realistic than a 10 or 30-minute protocol, where general anesthesia would likely be required. Increased or equivalent effectivity of an accelerated PACK-CXL treatment would make PACK-CXL much more accessible from both a clinical and client-driven perspective and requires further investigation.

The effect size (the magnitude of the OR) for the use of accelerated PACK-CXL protocols was reduced when an adjustment for the four different collaborating clinics was implemented in the model. The reason for the discrepancy in treatment success/failure across clinics is unknown. Hence, there may be additional factors influencing treatment outcome which were not included, such as the degree of severity of ulcers treated with PACK-CXL (early vs. late-stage disease), or clinician preferences in terms of PACK-CXL protocols used in the different clinics.

Although the collected information is deemed important by medical professionals, and the list of variables included is relatively comprehensive, certain crucial variables may not have been considered in the analysis. The authors acknowledge that including whether or not all patients required general anesthesia for PACK-CXL therapy would have been of interest to the reader, and this variable could be included in future studies. It is also possible that variables critical for treatment outcome were “hidden” under the exposure variable ‘acceleration’. Additionally, some necessary, but arguably arbitrary decisions for the categorisation of the exposure variables were made, due to the small number of eyes in the various subgroups. This data aggregation could have had an impact on the results. It must also be acknowledged that the group of patients that underwent a slow PACK-CXL protocol was much smaller than the fast protocol group (Figure S1), reflecting the unbalanced distribution of exposure variables in this dataset. This may result in a loss of statistical power, which, in addition to selection bias, is another common problem in retrospective studies.

The last aim of this study was to determine the proportion of treatment success in this population of dogs who received PACK-CXL as a component of their therapy. In the population of dogs evaluated in the present study, almost 90% experienced a successful outcome. This treatment success proportion is similar to the success proportion for PACK-CXL treatment in human patients with infectious keratitis reported in a meta-analysis and recent randomized controlled trial results paper [48, 49].

The survival analysis of the present study revealed that the use of an accelerated PACK-CXL protocol combined with topical therapy (a combination of antibiotics and anti-collagenolytics) shortened the corneal re-epithelialization time when compared to patients undergoing only PACK-CXL. As previously noted, the use of PACK-CXL as the sole treatment for infectious keratitis in veterinary species remains controversial. Therefore, until further prospective, randomized studies investigating the possible applications of PACK-CXL as a stand-alone therapy in veterinary species have been published, it would be prudent to combine its use with conventional topical therapies.

This study is, in part, a hypothesis-generating step, which might help guide the design of future RCTs regarding the selection of variables to be tested or controlled for. This is useful, as it is simply not possible to run RCTs that account for all possible risk and “success” factors. Despite the relative limitations of the present study design, the authors believe that the methodology presented here is still superior to a classical regression model. Additive Bayesian network analysis offers an added value for the analysis such as graphical representation of possible associations between variables, which may allow for an improved understanding of the connection between various factors and treatment success/failure. In the present study, ABN prevented the authors from drawing incorrect conclusions (which could have been made if based only on the regression model), thereby mitigating incorrect clinical recommendations.

Lastly, the authors wish to be clear that the focus of the present study was not to compare different treatment protocols. Instead, the aim was to evaluate whether recorded variables, including those associated with the PACK-CXL protocol utilized, might represent potential risk factors for treatment failure. Further, it should be noted that the number of patients in each treatment group was variable, and as such, the groups are unbalanced. It is therefore not possible to compare the different treatment protocols (something which can be done in an RCT), nor was that the goal of this study.

In conclusion, no risk factors associated with failure were identified in the population included in the present study. An accelerated (i.e., fast) PACK-CXL protocol might be associated with treatment success. The secondary analysis suggests that the use of an accelerated PACK-CXL protocol combined with the use of topical antibiotics and anti-collagenolytics shortened the corneal re-epithelialization time when compared to the use of PACK-CXL alone in the cases included here. Adequately powered, prospective, controlled, randomized studies with a broader scope of collected variables are needed to compare the effectiveness of PACK-CXL to that of other treatment options. As a hypothesis-generating study, the study presented here provides a rationale to guide the design of future randomized controlled trials regarding the selection of risk factors to be tested or controlled for.

### Electronic supplementary material

Below are the links to the electronic supplementary materials.

Supplementary Material 1

Supplementary Material 2

Supplementary Material 3

Supplementary Material 4

## Data Availability

Supplementary information is available including the raw data set, the cleaned data set, and the data management plan, which can be found on the OSF repository under the same title as the manuscript (osf.io/4hk6s). Mosaic plots and box plots, displaying the number of eyes in each subgroup from the variables included in the ABN, are available as supplementary materials (see Supplementary Figure S1). A summary of the raw data set is available as Table S3. Supplementary Figure S2 and Tables S1, S2 and S4 present the results of the generalised regression model and a summary of the exposure variables used in the Cox proportional hazard analysis.

## Abbreviations

AB: Antibiotic
ABN: Additive Bayesian network
AIC: Akaike information criterion
CI: Confidence interval
CrI: Credible interval
CXL: Corneal cross-linking
DAG: Directed acyclic graph
HPMC: Hydroxypropyl methylcellulose
HR: Hazard ratio
LRT: Likelihood ratio test
OD: Oculus dexter, right eye
OS: Oculus sinister, left eye
OSF: Open Science Framework
OR: Odds ratio
OU: Oculus uterque, both eyes
PACK-CXL: Photoactivated chromophore for keratitis – corneal cross-linking
RCT: Randomized controlled trial
