# Supplementary material for: Evaluation of risk factors for treatment failure in canine patients undergoing photoactivated chromophore for keratitis – corneal cross-linking (PACK-CXL): a retrospective study using additive bayesian network analysis
Source: BMC Vet Res. 2023 Nov 2;19:227. doi: 10.1186/s12917-023-03779-x (PMC10621152; doi:10.1186/s12917-023-03779-x)
Supplement: Supplementary file 1 — Supplementary Material 1 [file 12917_2023_3779_MOESM1_ESM.docx]

Data collection

**Purpose:** To determine risk factors that can serve as prognostic indicators of primary treatment failure among dogs presenting with presumed infectious keratitis who received PACK-CXL as a treatment.

**Design:** retrospective cross-sectional

**Analyses**:

- ABN - primary outcome
- Descriptive statistical analysis
- Separate survival analysis - secondary outcome

**Extracted Information:**

**##...##**- essential information for eligibility (needs to be provided for each included patient)

*******italic*****-** nice to have

**…-** nonessential

1. *##Demographics*: Species, Age, gender (male/female), breed, skull (brachy/mesocephalic)##
2. *Medical history*: ***duration of symptoms (duration of symptoms prior to PACK-CXL: time from symptoms to referral + time from first presentation to PACK-CXL Tx), previous/concurrent ocular morbidity and/or surgery (name condition or surgery), concurrent systemic morbidity (if possible name),*** recent topical/systemic AB, recent topical NSAIDs or corticosteroids (yes/no), recent systemic corticosteroids (yes/no).
3. *Ulcer*: ##size at presentation (mm2 or mm DM), depth (% of stromal loss), OS/OD/OU##; ***was culture performed (yes/no); culture results (positive, negative); culture results isolates, isolates susceptibility to AB treatment initiated or continued**;* Inflammatory infiltrate/Keratomalacia/Hypopyon (yes/no).
4. *Treatment:* date of visit*, ##*initial frequency and type of topical AB Tx initiated or continued by specialist. Type and frequency of anticollagenolytic(s) (topical and systemic)##, Systemic analgesia (yes/no)
5. Initial Tx plan: ##PACK-CXL (+ medical Tx) or PACK-CXL + surgical intervention##. Name of the surgical intervention that was performed as part of the initial Tx plan, PACK-CXL / surgery performed by trainee or senior ophthalmologist
6. *##PACK-CXL protocol*: Light source/equipment (name), fluence/total energy (J/cm^2^), irradiation intensity (mW/cm^2^), treatment time (min), Irradiation mode (continuous/pulsed/other), combination protocol details (for example: 9mW/cm^2^ x 10min + 45mW/cm^2^ x 2min + 45mW/cm^2^ x 2min), Chromophore solution (ribo concentration, carrier: Dextran/HPMC), Chromophore soak time/interval.##
7. ***In case of primary treatment failure: Rescue intervention: Type (globe saving Sx, Enucleation), Globe maintained post intervention?, Vision maintained post intervention?***

**Endpoints**

1. **Primary endpoint:** PACK-CXL Tx failure vs success. **Failure** defined as: necessity to change treatment to stabilize the cornea (including enucleation) = refractory to primary treatment
2. **Secondary endpoint**: days between PACK-CXL and Fluorescein negative stain

| **Risk factors- human literature** |
| --- |
| Significance demonstrated in human patient-based literature |
| - Ulcer size > 15mm^2^ [3, 5] (bacterial) |
| - Ulcer size > 14mm^2^ [1] (fungal) |
| - Central ulcer location[3] |
| - Deep infiltrate [1] (fungal) |
| - Limbal involvement [3] |
| - Hypopyon [1] (fungal) [2, 3] |
| - Age [3, 4] (bacteria) |
| - Culture positive [2] |
| - Previous corneal disease [2] |
| - History of ocular surgery[3] |
| - Topical steroids prior to presentation [3] |
| - Delay in referral [3] same in Acanthomoeba [6] |

***References***

- 1. Lalitha, P., et al., *Risk Factors for Treatment Outcome in Fungal Keratitis.* Ophthalmology, 2006. **113**(4): p. 526-530.
- 2. Morlet, N., D. Minassian, and J. Butcher, *Risk factors for treatment outcome of suspected microbial keratitis.* British Journal of Ophthalmology, 1999. **83**(9): p. 1027.
- 3. Miedziak, A.I., et al., *Risk factors in microbial keratitis leading to penetrating keratoplasty.* Ophthalmology, 1999. **106**(6): p. 1166-1171.
- 4. van der Meulen, I.J., et al., *Age-related Risk Factors, Culture Outcomes, and Prognosis in Patients Admitted With Infectious Keratitis to Two Dutch Tertiary Referral Centers.* Cornea, 2008. **27**(5).
- 5. Ahn, M., et al., *Clinical Aspects and Prognosis of Mixed Microbial (Bacterial and Fungal) Keratitis.* Cornea, 2011. **30**(4).
- 6. Bouheraoua, N., et al., *Prognostic Factors Associated With the Need for Surgical Treatments in Acanthamoeba Keratitis.* Cornea, 2013. **32**(2).
