# Supplementary material for: Evaluation of risk factors for treatment failure in canine patients undergoing photoactivated chromophore for keratitis – corneal cross-linking (PACK-CXL): a retrospective study using additive bayesian network analysis
Source: BMC Vet Res. 2023 Nov 2;19:227. doi: 10.1186/s12917-023-03779-x (PMC10621152; doi:10.1186/s12917-023-03779-x)
Supplement: Supplementary file 2 — Supplementary Material 2 [file 12917_2023_3779_MOESM2_ESM.docx]

**Detailed methods, ABN analysis:**

ABN results consist of two components. The first component is structural, resulting in the creation of directed acyclic graphs (DAG). A DAG can be understood as a map of variables and existing associations. In a DAG, variables are visualized as nodes, and two nodes can be linked to each other by an arc, which represents the association between two nodes/variables. In this association one node is called a parent and the other a child.

The ABN analysis was performed using a two-step approach. In the first step, the optimal level of complexity was established by increasing the number of parents linked directly to the child. In the second step, the model was checked for robustness and potential overfitting with a Markov Chain Monte Carlo (MCMC) simulation over the structure [30]. We evaluated the posterior distributions to check model robustness. For the MCMC simulation, we used a thinning factor of 10, and a burn-in phase of 25% of the total number of MCMC iterations. The final DAG was determined by removing any arcs supported by < 50 % of the 1000 DAGs. The strength of each arc was quantified using link strength (LS) and is reflected in the arc thickness in the DAG. ABN analyses, including LS calculation and MCMC simulations, were implemented using the “abn” [31] and “mcmcabn” [32] packages, respectively.

**Detailed methods, Survival Analysis:**

The validity of the proportional hazards’ assumptions were tested with proportional hazards tests (cox.zph from survival package [34]) and linearity by plotting martingale residuals against fitted values.

Variables for the model were selected based on a standard step-wise selection process. We considered 18 exposure variables. In brief, in the first stage, a likelihood ratio test (LRT) was used to compare the model without (null model) and with a variable, to evaluate whether a model with variable is significantly better than a null model. This step was repeated for each variable. A variable was included in subsequent steps if the p-value calculated with the LRT was < 0.2. In the second stage, complex models including a combination of variables were compared with LRT and Akaike information criterion (AIC). The simplest model was favored. Based on this approach, age, skull type, time until referral, AB prior, steroids prior, systemic disease, nasolacrimal disease, ulcer depth, keratomalacia, riboflavin carrier and riboflavin concentration, and fluence were not significant and therefore not included in the Cox proportional hazard regression.
