# Supplementary material for: Evaluation of risk factors for treatment failure in canine patients undergoing photoactivated chromophore for keratitis – corneal cross-linking (PACK-CXL): a retrospective study using additive bayesian network analysis
Source: BMC Vet Res. 2023 Nov 2;19:227. doi: 10.1186/s12917-023-03779-x (PMC10621152; doi:10.1186/s12917-023-03779-x)
Supplement: Supplementary file 3 — Supplementary Material 3 [file 12917_2023_3779_MOESM3_ESM.pdf]

Figure S1. Mosaic and box plots displaying the number of eyes in each subgroup from the variables included in the ABN.

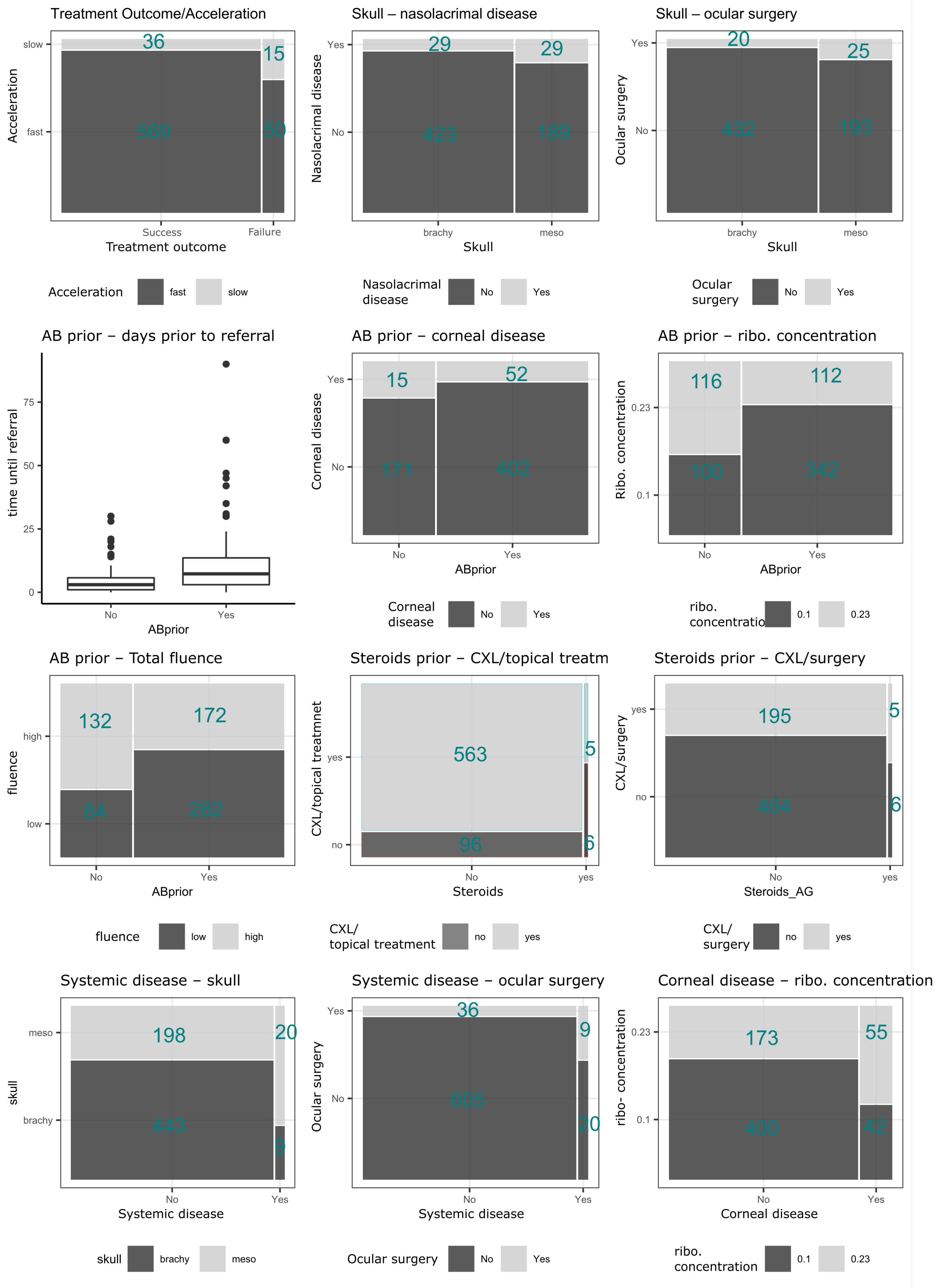

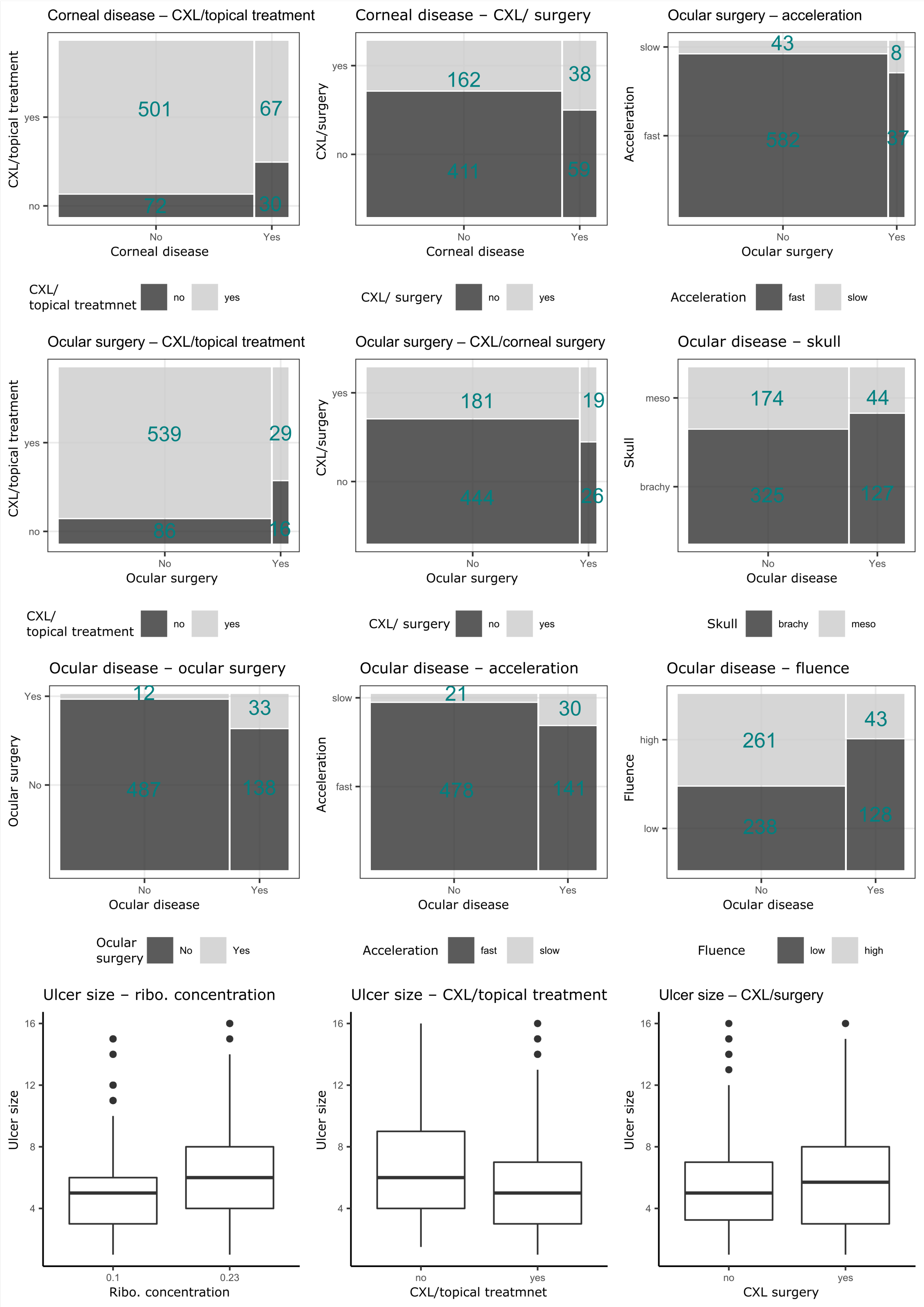
Corneal disease – CXL/topical treatment
CXL/topical treatment
yes
no
501
67
72
30
No
Yes
Corneal disease
CXL/
topical treatmnet
no
yes
Corneal disease – CXL/ surgery
CXL/surgery
162
38
411
59
Corneal disease
CXL/ surgery
Ocular surgery – acceleration
Acceleration
slow
fast
43
8
582
37
Ocular surgery
fast
slow
Ocular surgery – CXL/topical treatment
CXL/topical treatment
539
29
86
16
Ocular surgery
CXL/
topical treatment
Ocular surgery – CXL/corneal surgery
CXL/surgery
181
19
444
26
Ocular surgery
CXL/ surgery
Ocular disease – skull
Skull
meso
brachy
174
44
325
127
Ocular disease
Skull
Ocular disease – ocular surgery
Ocular surgery
12
33
487
138
Ocular disease
Ocular
surgery
Ocular disease – acceleration
Acceleration
21
30
478
141
Ocular disease
Ocular disease – fluence
Fluence
high
low
261
43
238
128
Ocular disease
Ulcer size – ribo. concentration
Ulcer size
16
12
8
4
0.1
0.23
Ribo. concentration
Ulcer size – CXL/topical treatment
CXL/topical treatmnet
Ulcer size – CXL/surgery
CXL surgery

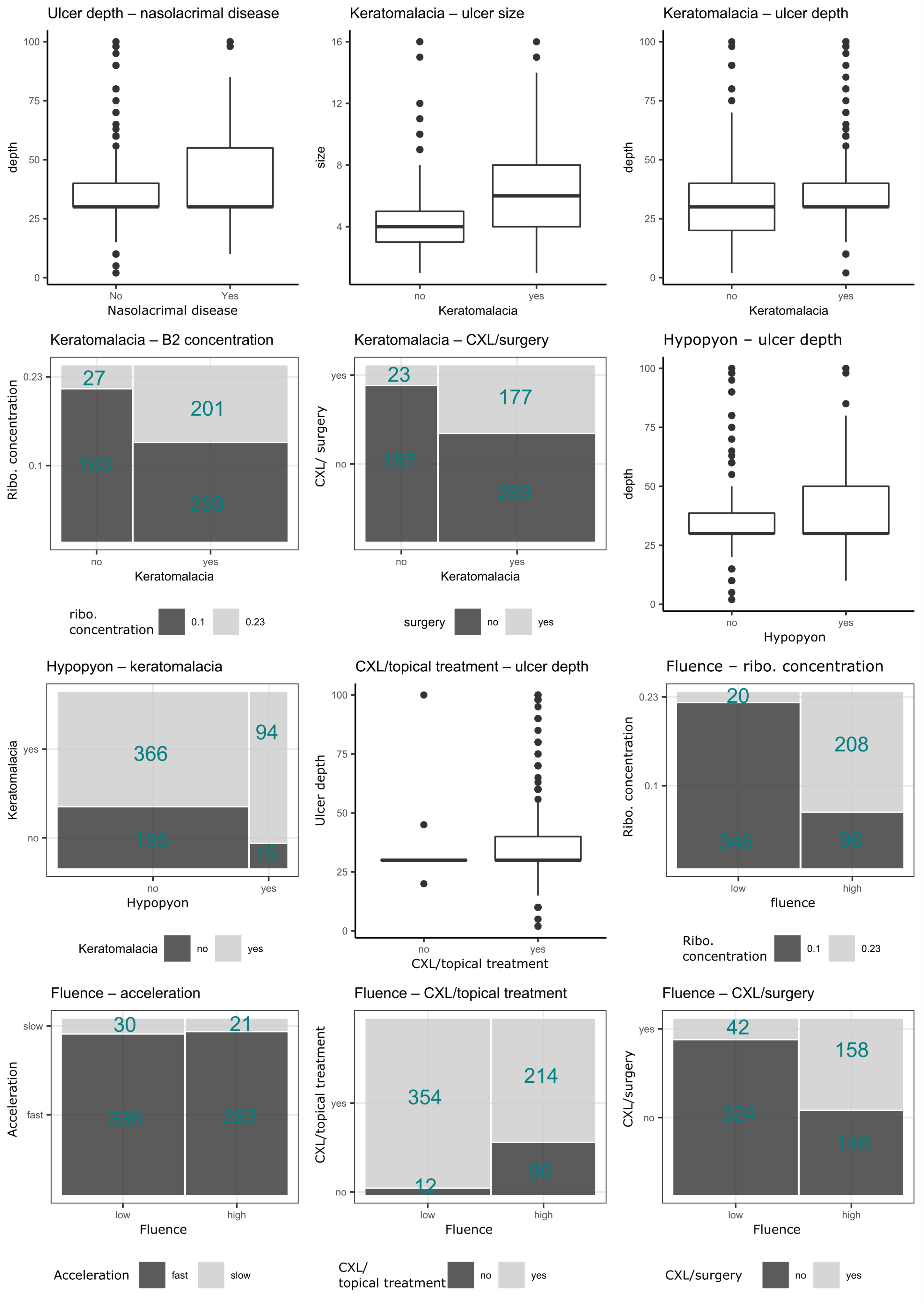

Ulcer depth – nasolacrimal disease
depth
100
75
50
25
0
No
Yes
Nasolacrimal disease
Keratomalacia – ulcer size
size
16
12
8
4
no
yes
Keratomalacia
Keratomalacia – ulcer depth
depth
100
75
50
25
0
no
yes
Keratomalacia
Keratomalacia – B2 concentration
Ribo. concentration
0.23
0.1
27
201
183
259
no
yes
Keratomalacia
ribo. concentration
0.1
0.23
Keratomalacia – CXL/surgery
CXL/ surgery
yes
no
23
177
187
283
no
yes
Keratomalacia
surgery
no
yes
Hypopyon – ulcer depth
depth
100
75
50
25
0
no
yes
Hypopyon
Hypopyon – keratomalacia
Keratomalacia
yes
no
366
94
195
15
no
yes
Hypopyon
Keratomalacia
no
yes
CXL/topical treatment – ulcer depth
Ulcer depth
100
75
50
25
0
no
yes
CXL/topical treatment
Fluence – ribo. concentration
Ribo. concentration
0.23
0.1
20
208
346
96
low
high
fluence
Ribo. concentration
0.1
0.23
Fluence – acceleration
Acceleration
slow
fast
30
21
336
283
low
high
Fluence
Acceleration
fast
slow
Fluence – CXL/topical treatment
CXL/topical treatment
yes
no
354
214
12
90
low
high
Fluence
CXL/ topical treatment
no
yes
Fluence – CXL/surgery
CXL/surgery
yes
no
42
158
324
146
low
high
Fluence
CXL/surgery
no
yes

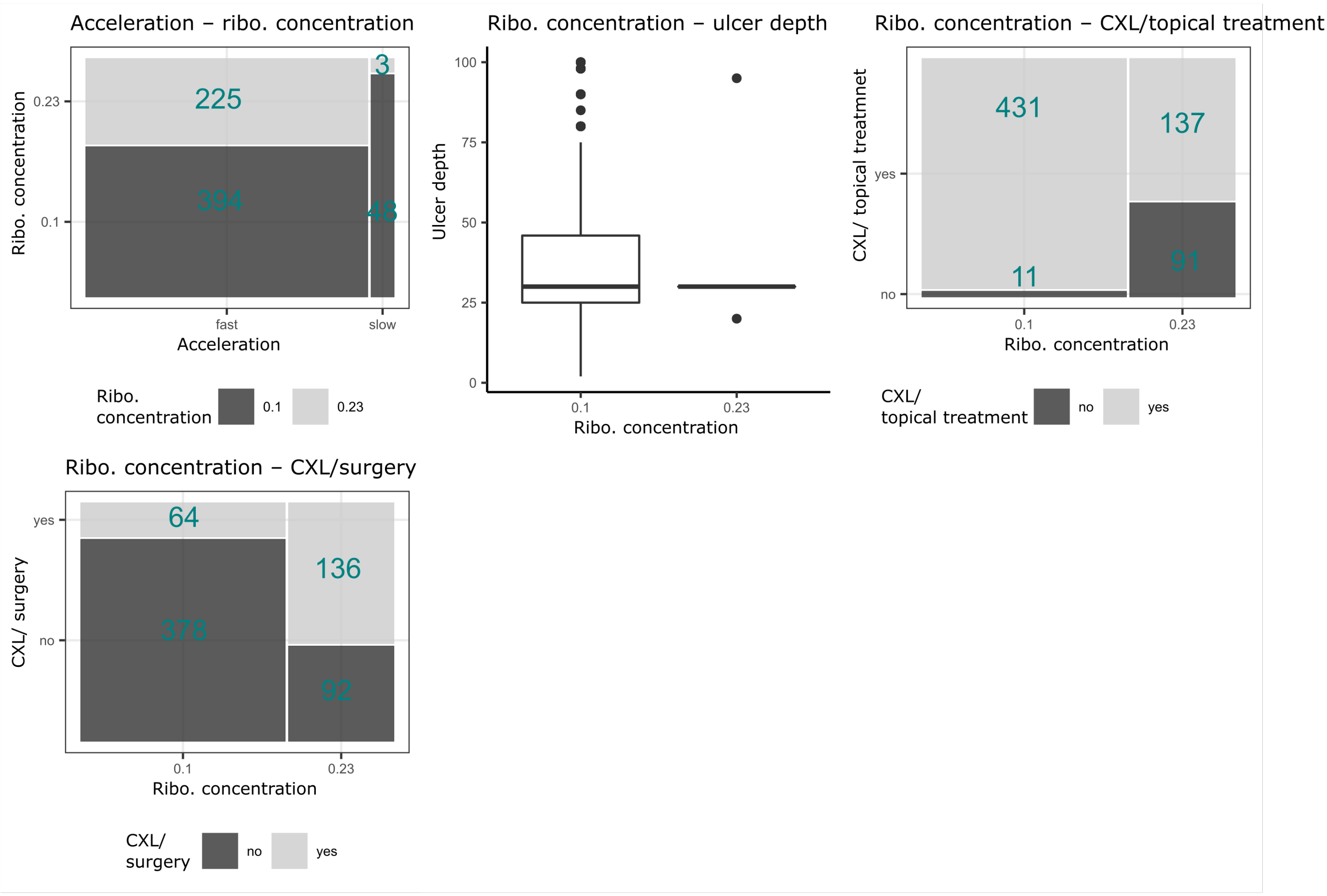

Legend: In a mosaic plot the width of the columns is proportional to the number of observations in each level of the variable plotted on the horizontal axis. The vertical length of the bars is proportional to the number of observations in the second variable within each level of the first variable.
