# Supplementary material for: Evaluation of risk factors for treatment failure in canine patients undergoing photoactivated chromophore for keratitis – corneal cross-linking (PACK-CXL): a retrospective study using additive bayesian network analysis
Source: BMC Vet Res. 2023 Nov 2;19:227. doi: 10.1186/s12917-023-03779-x (PMC10621152; doi:10.1186/s12917-023-03779-x)
Supplement: Supplementary file 4 — Supplementary Material 4 [file 12917_2023_3779_MOESM4_ESM.pdf]

Figure S2. DAG created based on the regression results.

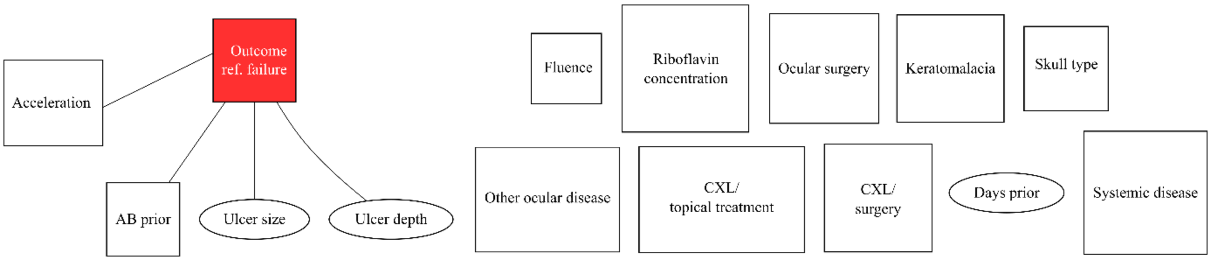

It is possible only to indicate directly associated variables, other associations not identified (variables not connected to the outcome).

**Table S1**. Medical history data used in the Cox proportional hazard analysis. Summary of categorical variables in dogs.	
	n (%), [95%CI]
Treatment outcome	
Failure	423 (98), [96 to 99]
Success	10 (2), [0.8 to 4]
Patient demographics	
Skull type	
brachycephalic	280 (65), [60 to 69]
mesocephalic	153 (35), [30 to 40]
Medical history	
AB prior	
No	117 (27), [23 to 31]
Yes	316 (73), [69 to 77]
Systemic disease	
No	417 (96), [94.5 to 98]
Yes	16 (4), [2 to 5]
Steroids prior	
No	426 (98), [97.5 to 99.7]
Yes	6 (1), [0.2 to 2.5]
Corneal disease	
No	379 (87.5), [84 to 90]
Yes	54 (12.5), [9 to 15.5]
Ocular surgery	
No	410 (95), [92.5 to 97]
Yes	23 (5), [3 to 7]
Nasolacrimal disease	
No	402 (93), [90 to 95]
Yes	31 (7), [5 to 9.5]
Other ocular disease	
No	307 (71), [66 to 75]
Yes	126 (29), [25 to 33]
Corneal ulcer parameters	
Keratomalacia	
No	182 (42), [37 to 46]
Yes	251 (58), [53 to 63]
Hypopyon	
No	364 (84), [80 to 87.5]
Yes	69 (16), [12.5 to 19]
Treatment plan	
CXL/topical treatment	
No	91 (21), [17 to 25]
Yes	342 (79), [75 to 83]
CXL/surgery	433 (100)
No	
PACK-CXL protocol parameters	
Fluence (J/cm^2)	
5.4	301 (69.5), [65 to 74]
10.8	22 (5), [3 to 7]
16.2	110 (25.5), [21 to 29.5]
Acceleration	
Fast	403 (93), [91 to 95.5]
Slow	30 (7), [4.5 to 9]

Riboflavin concentration	
0.1%	351 (81), [77 to 85]
0.23%	79 (18), [15 to 22]
0.25%	3 (0.7), [0 to 1.5]
**Total**	433

Table S2 Medical history data used in the Cox proportional hazard analysis. Summary of continuous variables in dogs.

	**Total**<br>Medium (interquartile range)
Age (years)	7 (4 to 10)
Time until referral (days)	55 (5 to 80)
Corneal ulcer parameters	
Ulcer size (mm)	5 (2 - 7)
Ulcer depth (%)	30 (30 - 40)

**Table S3** Data summary before imputation of missing values.				
A) Number of dog eyes in each category for exposure variables.				
ID		Treatment **success** group n (%)	Treatment **failure** group n (%)	Total n (%)
	Patient demographics			
1	Skull type			
	brachycephalic	406 (90)	47 (10)	453 (67.5)
	mesocephalic	199 (91)	19 (9)	218 (32.5)
	Medical history			
2	AB prior			
	No	190 (87.5)	27 (12.5)	217 (32)
	Yes	400 (92)	36 (8)	436 (65)
	Missing	15	3	18 (3)
3	Systemic disease			
	No	551 (92)	46 (8)	597 (89)
	Yes	24 (83)	5 (17)	29 (4)
	Missing	30	15	45 (7)
4	Steroids prior			
	No	597 (90.5)	63 (9.5)	660 (98)
	Yes	8 (73)	3 (27)	11 (2)
	Missing	0	0	0
5	Corneal disease			
	No	514 (90.5)	54 (9.5)	568 (85)
	Yes	85 (88)	12 (12)	97 (14)
	Missing	6	0	6 (1)
6	Ocular surgery			
	No	562 (91)	58 (9)	620 (92)
	Yes	37 (82)	8 (18)	45 (7)
	Missing	6	0	6 (1)
7	Nasolacrimal disease			
	No	547 (90)	60 (10)	613 (90.5)
	Yes	52 (90)	6 (10)	58 (8)
	Missing	6	0	6 (1)
8	Other ocular disease			
	No	456 (91)	43 (9)	499 (74)
	Yes	143 (86)	23 (14)	166 (25)
	Missing	6	0	6 (1)
	Corneal ulcer parameters			
9	Keratomalacia			
	No	198 (94)	12 (6)	210 (31)
	Yes	405 (89)	52 (11)	457 (68)
	Missing	2	2	4 (1)
10	Hypopyon			
	No	505 (91)	50 (9)	555 (83)
	Yes	95 (87)	14 (13)	109 (16)
	Missing	5	2	7 (1)
	Treatment plan			

11	CXL/topical treatment			
	No	88 (86)	14 (14)	102 (15)
	Yes	517 (91)	52 (9)	569 (85)
	Missing	0	0	0
	CXL/surgery			
12	No	423 (90)	47 (10)	470 (70)
	Yes	182 (91)	19 (9)	201 (30)
	Missing	0	0	0
	PACK-CXL protocol parameters			
13	Fluence (J/cm^2)			
	5.4	333 (91)	33 (9)	366 (54.5)
	10.8	38 (97.5)	1 (2.5)	39 (6)
	16.2	233 (88)	31 (12)	264 (39)
	≥21.6	1 (50)	1 (50)	2 (0.5)
	Missing	0	0	0
14	Acceleration			
	Fast	568 (92)	51 (8)	619 (92)
	Slow	36 (70.5)	15 (29.5)	51 (7.5)
	Missing	1	0	1 (0.5)
15	Riboflavin concentration			
	0.1%	399 (91)	40 (9)	439 (65)
	0.23%	173 (90)	19 (10)	192 (29)
	0.25%	4 (100)	0	4 (1)
	Missing	29	7	36 (5)
	**Total**	605 (90) [88 to 92]	66 (10) [7 to 12]	671 (100)
**B)** Medical history data and continuous variables				
ID		Treatment **success** group median (interquartile range)	Treatment **failure** group median (interquartile range)	**Total** median (interquartile range)
	Medical history			
16	Time until referral (days)	6 (3 - 10)	3 (1 - 8)	5 (2 - 11)
	Missing	n=104	n=12	n=116
	Corneal ulcer parameters			
17	Ulcer size (mm)	5 (3 - 7)	6 (4 - 8)	5 (3 - 7)
	Missing	n=7	n=5	n=12
18	Ulcer depth (%)	30 (30 - 40)	30 (30 - 40)	30 (30 - 40)
	Missing	n=4	n=6	n=10

**Table S4.** Results of the classical mixed model regression analysis			
Exposure Variable	*P value*	*Effect size*	*95% Confidence Intervals*
Acceleration	0.0496	3.7	0.94 to 10.83
AB prior	0.032	0.52	0.28 to 0.95
Ulcer size	0.0239	1.09	1.01 to 1.19
Ulcer depth	0.0453	1.01	1 to 1.03
